# Age-Related Macular Degeneration: A Disease of Systemic or Local Complement Dysregulation?

**DOI:** 10.3390/jcm3041234

**Published:** 2014-11-03

**Authors:** Alasdair Warwick, Samir Khandhadia, Sarah Ennis, Andrew Lotery

**Affiliations:** 1 Clinical Neurosciences Research Group, Clinical and Experimental Sciences, Faculty of Medicine, University of Southampton, Southampton SO16 6YD, UK; E-Mail: alasdair.warwick06@gmail.com; 2 Eye Unit, University Southampton NHS Trust, Southampton SO16 6YD, UK; E-Mail: sam.khandhadia@nhs.net; 3 Genomic Informatics, Human Genetics & Genomic Medicine, Faculty of Medicine, University of Southampton, Southampton SO16 6YD, UK; E-Mail: s.ennis@soton.ac.uk

**Keywords:** age-related macular degeneration, complement pathway, complement system proteins/genetics, pathway analysis, proteomics

## Abstract

Age-related macular degeneration (AMD) is the leading cause of irreversible blindness in developed countries. The role of complement in the development of AMD is now well-established. While some studies show evidence of complement dysregulation within the eye, others have demonstrated elevated systemic complement activation in association with AMD. It is unclear which one is the primary driver of disease. This has important implications for designing novel complement-based AMD therapies. We present a summary of the current literature and suggest that intraocular rather than systemic modulation of complement may prove more effective.

## 1. Introduction

Age-related macular degeneration (AMD) is a common disease of the elderly and the leading cause of irreversible visual loss in the developed world [1]. Early AMD is characterised by the appearance of pigmentary changes and drusen in the retina. Loss of central vision occurs with disease progression either due to atrophy of the retinal pigment epithelial (RPE) cell layer and photoreceptors in geographic atrophy (GA), or haemorrhage or fluid exudation from choroidal neovascularisation (CNV) in neovascular (NV) AMD. GA is associated with gradual reduction in vision, whereas NV causes acute visual loss. While intravitreal injections of anti-vascular endothelial growth factor (VEGF) have revolutionised the treatment of NVAMD, an effective treatment for GA remains elusive [2].

The pathogenesis of AMD is complex and multifactorial. Age, environmental factors, genetic predisposition and oxidative stress are all thought to contribute [2]. An inflammatory model of AMD proposes that complement dysregulation can initiate and potentiate local inflammation at the retina in individuals with certain genetic and environmental risk factors [3]. Evidence for the role of complement, part of the innate immune system, in AMD began with the discovery that drusen contains various complement components and their regulators [4,5,6,7,8]. Such observations led to the concept that drusen, the clinical hallmark of early AMD, may act as foci of retinal inflammation. Following this in 2005, landmark studies demonstrated an association between a polymorphism in the gene encoding complement factor H (CFH) and AMD development [9,10,11,12]. Several further risk variants in complement-related genes have since been discovered, including *C2*/*CFB* [13], *C3* [14], possibly *C7* [15], *C9* [16], *CFI* [17] and *SERPING* [18], although some controversy exists over the significance of the latter [19]. The importance of these genes is supported by features exhibited by gene-specific knock-out mice. For example, *CFH*−/− mice show significantly reduced electroretinogram responses, increased subretinal fluorescent material, and disruption of RPE and photoreceptor cells, suggesting a protective role for *CFH* [20]. *C3*−/− [21] and *C5*−/− [22] mice display increased resistance to the development of laser-induced choroidal neovascularisation (CNV). In addition, a number of studies have demonstrated increased systemic complement activity in AMD patients compared with healthy controls [23,24,25,26,27,28,29,30,31,32]. 

While altered complement deposition within the eye and increased systemic complement activation have both been demonstrated in AMD, it is unclear which one is the primary driver of disease. Clarifying this issue has important implications for designing novel complement-based therapies. This review provides an overview of the complement system, followed by a summary of the evidence for local and systemic dysregulation in AMD, and ends by comparing whether local intraocular or systemic complement activity is more important in AMD.

## 2. The Complement System: An Overview

**Figure 1:**
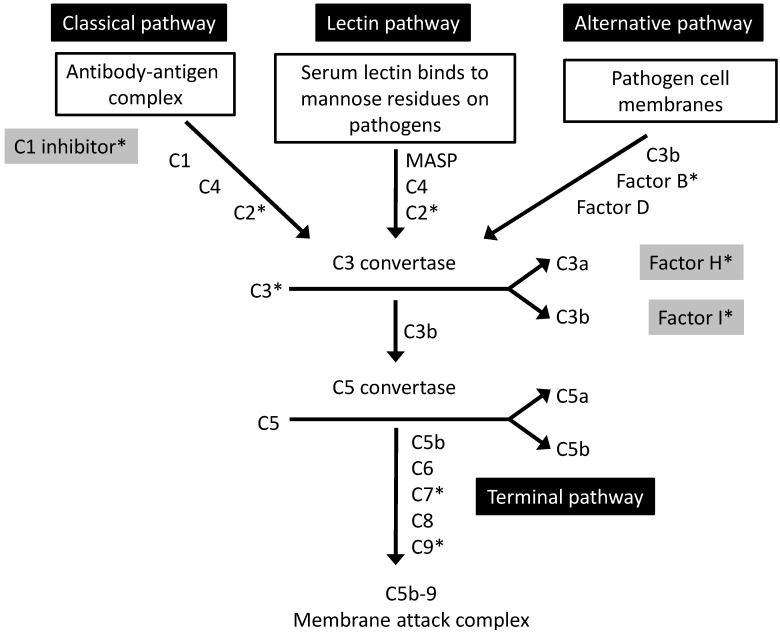
The complement cascade. Complement regulators are shown in grey boxes. Complement components and regulators which have known genetic associations with AMD are marked with an asterisk (*). Adapted from Khandhadia *et al.* 2012 [33].

The complement system encompasses a family of more than 30 circulating proteins and their regulators, playing a key role in host defence through pathogen recognition, opsonisation and lysis. It also performs important immunoregulatory functions, clearing immune complexes, inflammatory products and apoptotic cells, as well as bridging innate and adaptive immunity. Activation via the classical, lectin or alternative pathways triggers a sequential amplifying cascade of enzymatic reactions (Figure 1). All three pathways converge with the production of a C3 convertase, which in turn produces a C5 convertase. This then activates the terminal pathway, culminating with formation of the membrane attack complex (MAC). The other effectors of the complement cascade are the anaphylatoxins, of which C5a is the most potent inflammatory mediator, and opsonins such as C3b.

The classical pathway is initiated upon binding of C1q to complement-fixing antibodies in immune complexes. Antibody-bound C1q is able to activate C1r and C1s. C2 and C4 are subsequently cleaved by activated C1s, producing C2a, C2b, C4a and C4b. The larger C4b and C2a fragments combine to form a C3 convertase (C4bC2a) which can cleave C3 into C3a and C3b. C3b binds to C3 convertase to form a C5 convertase (C4bC2aC3b) which cleaves C5 into C5a and C5b. C5b associates non-enzymatically with C6-C9, forming the MAC (C5b-C9). The MAC induces cell lysis by forming a pore-like structure in the phospholipid bilayer.

Binding of mannan-binding lectin (MBL) to carbohydrate ligands on microbial cell surfaces activates the lectin pathway. MBL-associated serine proteases (MASP 1, MASP-2 and MASP-3) bind to these pattern-recognition molecules and the MBL-MASP-2 complex activates C4 and C2. C3 convertase (C4bC2a) is formed and the complement cascade proceeds in the manner of the classical pathway thereafter.

In contrast to the classical and lectin pathways, the alternative pathway continuously self-activates at a low level via spontaneous hydrolysis of C3 into C3a and C3b. C3b binds to factor B (CFB), allowing factor D (CFD) to cleave CFB into Ba and Bb fragments. Bb remains attached to C3b to create C3bBb, the alternative pathway C3 convertase. This can cleave further C3 into C3a and C3b, forming the basis of an amplification loop. C3bBb binds to C3b to form C3bBb3b, the alternative pathway C5 convertase. C3bBb3b converts C5 to C5a and C5b, leading to initiation of the terminal pathway.

Numerous soluble and membrane-bound regulators are required to prevent uncontrolled complement activation. These act by degrading complement components and increasing convertase decay or by inhibiting MAC assembly. CFH is the most important fluid phase regulator and the main inhibitor of the alternative pathway. CFH catabolises C3b and accelerates C3 convertase decay. Complement factor I (CFI) degrades C3b and C4b, requiring cofactors such as CFH, membrane cofactor protein (MCP) or complement receptor 1. For further information on the complement system and its regulation, the reader is referred to the following reviews [34,35,36].

The complement system therefore exists in a delicate homeostatic balance between destroying pathogens and minimising damage to local tissues. Dysregulation is associated with a variety of diseases including SLE [37], atypical haemolytic uraemic syndrome [38], dense deposit disease [39], as well as AMD [33]. While increased complement activity may be beneficial for protection against infection in early life, chronic low-grade inflammation may prove detrimental in susceptible tissues with increasing age [40,41]. AMD studies have consistently implicated involvement of the alternative pathway in particular. As discussed above, the alternative pathway of complement activation is both spontaneously activating and self-perpetuating, providing a potential explanation for how complement could both initiate and amplify such inflammation.

## 3. Intraocular Complement and AMD

Intraocular complement production alters with age and in AMD. This may result in complement overactivity and contribute to retinal damage in AMD. The following section reviews the evidence for this process and the potential pathogenic mechanisms by which it may occur.

### 3.1. Complement Production in the Ageing Retina

While the majority of circulating complement is produced by the liver, a variety of tissues demonstrate extrahepatic complement synthesis [42]. The retina is one such example with production of some components approaching hepatic levels [9]. Anderson *et al.* showed using quantitative PCR that the human retina expresses a wide range of complement components and regulators, particularly those of the classical and alternative pathways. The choroid was the main source of complement proteins, whereas expression by the RPE and neural retina was mostly limited to a subset of alternative and terminal pathway regulators [3]. Resident microglia have also been recognised as a source of local complement. Luo *et al.* found that *in vitro* mouse retinal microglia constitutively produce complement, which is regulated by inflammatory cytokines interferon-gamma (IFNγ) and tumour necrosis factor-alpha (TNFα) [43].

Extrahepatic complement production may be necessary in tissues that are particularly vulnerable to infection or where delivery of circulating hepatic complement is limited [44]. The blood-retinal barrier restricts access of plasma proteins to the eye and may explain the need for supplemental local production. Recent evidence also suggests a physiological role for complement in retinal function and neuronal survival [45]. Yu *et al.* showed that mice lacking receptors for C3a and C5a developed progressive early-onset retinal degeneration and were more susceptible to light-induced retinal dysfunction compared with wild type controls [46]. Hoh Kam *et al.* found more pronounced photoreceptor loss and Bruch’s membrane thickening in aged *C3*−/− mice compared with wild type controls, suggesting a protective role for C3 in the aged retina [47].

Conversely it has been proposed that while local complement synthesis may be beneficial for early survival, it could theoretically increase susceptibility to damage from chronic low-grade overactivity in later years of life [3]. Indeed retinal production and deposition of complement appears to increase with advancing age. In studies of human donor eyes, immunohistochemistry showed that the majority of older subjects had MAC deposits in the RPE-choroid whereas this was rarely detectable in younger eyes [9,48]. Further evidence comes from studies on mice of differing ages. Mandal *et al.* showed greater expression of CFH in aged mice compared with younger mice [49]. Chen *et al.* also found that expression of C1q, C1r, C1s, C3 and CFB, as well as levels of C1q and C3 deposition in the RPE/choroid complex increased with age in mice [50]. A later study by the same authors suggested that increased complement activation might extend to the neuroretinal layer, as evidenced by increased deposition of the complement activation product C3d in the neuroretinas of older mice [51]. Similar results are reported by Faber *et al*., who in addition demonstrated higher expression of complement regulators CD59a and CFH in the neuroretinas of older mice [52]. More recently, intraretinal microglia extracted from older rats have been shown to express increased levels of C3 and CFB [53,54]. Expression of both complement components and their regulators therefore appear to increase with age. One possible explanation is that upregulation of complement regulators occurs in response to greater complement activity, the balance between these two processes determining susceptibility to complement-mediated retinal damage.

### 3.2. Complement Production and Deposition in AMD

Studies in mouse models suggest altered local complement production also occurs in AMD. Using reverse transcriptase PCR, upregulated retinal C1qβ [55] and C3 [56] expression has been shown in light-induced models of retinal degeneration. C3 expression was also upregulated in a laser-induced CNV model [57]. In addition, Bora *et al.* found upregulated local CFB expression in laser-induced CNV [22]. The same group also reported an initial decrease in CFH expression, which then increased again by day five post-laser [22,58]. CD59 followed a similar pattern, showing decreased expression following laser before increasing again by one week [59]. These findings collectively indicate a role for locally produced complement in retinal disease. The importance of retinal complement was perhaps most clearly demonstrated by Lyzogubov *et al.* Mice receiving subretinal injections of siRNA CFH showed a threefold reduction in retinal CFH production. Although hepatic levels of CFH and systemic alternative pathway activity remained unaltered, early onset and exacerbation of laser-induced CNV was observed in these mice compared with controls [58].

In human studies, immunohistochemical and proteomic studies have revealed differences in complement deposition comparing eyes from AMD patients with controls. Complement components and regulators are a prominent component of drusen [4,5,6,7,8] and have also been found in surgically removed CNV [60,61,62]. Further to this, quantitative proteomics analysis of AMD cadaveric eyes has shown elevated levels of complement proteins in the macular Bruch’s membrane/choroidal complex compared with controls [63]. Another group found C3, CFB and CFD were elevated in the Bruch’s membrane/choroid interface at the macula in advanced AMD compared with early disease. Analysis of vitreous samples further revealed increased CFB activation in more advanced disease, which was attributed to a combination of *CFH*, *C2*, *CFB* and *C3* genetic variants [64]. Homozygosity for the AMD-associated *CFH* Y402H polymorphism is also associated with increased MAC deposition at the RPE/choroid [65].

Other studies have found reduced levels of complement regulators in AMD donor eyes. Mullins *et al.* reported increased choroidal abundance of C1 inhibitor in AMD donor eyes compared with controls [66]. MCP is a membrane-bound complement regulator that has cofactor activity for CFI, acting at the convertase level of the complement cascade by inactivating C3b and C4b. Expression is normally on the basolateral surface of human RPE cells. Vogt *et al.* showed a reduction of MCP in early geographic atrophy, which was also associated with loss of polarity [67]. A more recent study additionally found reduced levels of CD59, a membrane-bound inhibitor of MAC formation, on RPE cells overlying drusen and geographic atrophy in AMD patients [68]. Interestingly CFH deposition in the retina is consistently greater in AMD compared to controls [9,69,70,71]. Perhaps this may reflect its importance in countering complement overactivation. Indeed, Hageman *et al.* showed that CFH co-localises with its ligand C3b/iC3b in drusen and co-distributes with MAC at the RPE/choroid [9].

### 3.3. Complement Activation and AMD: Pathogenic Mechanisms

Intraocular complement activity therefore appears to increase with advancing age and in AMD. Elucidating the underlying reasons for this process is the subject of much current research. Some studies have examined how AMD-associated genetic variants, in particular the *CFH* Y402H variant, may alter complement function and therefore predispose to greater activity. Other investigations have revealed potential triggers for retinal complement activation including oxidative stress, pro-inflammatory agents produced in the retina and amyloid beta. These findings are discussed below.

#### 3.3.1. Functional Consequences of the CFH Y402H Genetic Variant

The non-synonymous *CFH* Y402H polymorphism was the first complement-related genetic variant to be associated with AMD [9,10,11,12]. As a consequence, this has been the most extensively investigated variant in functional studies. However, it is not known whether the *CFH* Y402H variation has a direct influence on the pathogenesis of AMD, or whether it is a marker of an as yet unknown, perhaps more distal genetic defect.

The CFH protein is a key plasma regulator of C3b, the central component in the alternative pathway of complement activation. It consists of 20 short complement regulatory (SCR) domains that represent binding sites for ligands including C3b, C-reactive protein (CRP) and heparin [72]. By recognising host tissues through interactions with surface polyanions it can deactivate deposited C3b [73]. SCRs 1–4 are involved in C3b binding, decay accelerating and CFI cofactor activity, hence regulating C3 convertase activity. SCRs 12–14 and 19–20 bind to the C3b proteolytic fragments C3c and C3d respectively [74].

The risk *CFH* Y402H polymorphism leads to an amino acid change at position 402 of the CFH polypeptide, substituting tyrosine (Y402) for histidine (H402). Whereas other non-synonymous AMD-associated polymorphisms have been shown to directly affect alternative pathway activity [16,75,76,77], position 402 of the CFH protein lies within SCR7, which binds to streptococcal M6 protein, CRP and heparin [78]. CFH may therefore be especially important in protecting extracellular matrix structures like Bruch’s membrane, which do not express membrane-bound complement regulatory proteins. Clark *et al.* showed impaired binding of the AMD-associated H402 variant to glycosaminoglycans in Bruch’s membrane [79]. However, this was not replicated by Kelly *et al.* [80]. The H402 variant has also been found to bind less strongly to RPE cells by some studies [81,82], but again not by others [79,83].

A more consistent finding is that the CFH H402 variant shows reduced affinity for CRP, an acute phase protein produced by the liver as part of the inflammatory response [81,83,84,85,86,87,88]. CRP associates with numerous ligands, allowing it to bind to the surface of damaged or apoptotic cells [89]. It can also bind CFH and may therefore recruit CFH to damaged cells [82]. Johnson *et al.* found homozygosity for CFH H402 to be associated with elevated levels of CRP in the RPE/choroid of human donor eyes, although not with local CFH protein levels. The authors concluded this could reflect reduced ability of the CFH H402 allotype to bind CRP at the retina [70]. This may result in less complement regulation and consequently greater local inflammation. Furthermore, CRP can interact with C1q to activate the classical complement pathway and may also therefore contribute to local inflammation [90]. Indeed higher levels of circulating CRP are associated with a greater risk of late-onset AMD development [91] and AMD progression [92]. In addition, elevated systemic CRP levels and homozygosity for CFH H402 synergistically increase the risk of late AMD and disease progression [93]. Bhutto *et al.* reported higher levels of CRP in eyes with early or NV AMD, as well as in non-atrophic areas of eyes with GA. In contrast to the findings from Johnson *et al.* [70], significantly lower levels of CFH were observed in these eyes, suggesting an inverse relationship between CFH and CRP levels in AMD [94]. Interestingly, both CRP and CFH levels were lower in atrophic areas of GA however [94], which the authors attributed to lower vascular supply at these regions [95].

The CFH H402 protein variant also appears to be less protective against certain retinal mediators of oxidative stress. Weismann *et al.* investigated the interaction between CFH and malondialdehyde (MDA), a reactive decomposition product generated by lipid peroxidation of membrane phospholipids. CFH was shown to bind MDA and inactivate complement on MDA-bearing surfaces. However, this effect was diminished with the CFH H402 variant [69]. Oxidised phospholipids (oxPLs) represent another source of retinal oxidative stress. OxPLs are present in RPE cells and photoreceptors at the normal human macula and their levels have been shown to increase with age. Furthermore, eyes from AMD patients showed more intense immunoreactivity for oxPLs than age-matched control eyes [96]. Shaw *et al.* found that the protective CFH Y402 variant displayed greater binding to oxPLs and was more able to inhibit inflammatory effects on RPE cells and macrophages. By further showing that subretinal injections of oxPLs can induce CNV in mice, the authors concluded the CFH H402 variant may increase AMD-susceptibility through impaired ability to bind oxPLs [71].

#### 3.3.2. Potential Triggers for Complement Activation in AMD

##### 3.3.2.1. Oxidative Stress

Oxidative stress is recognised as an important aetiological factor in AMD pathogenesis [2]. A number of studies suggest that oxidative stress may cause retinal damage through local dysregulation of complement. For example, IFNγ-induced synthesis of CFH by cultured RPE cells is reduced following treatment with hydrogen peroxide [97] and exposure to blue light [98]. Ebrahimi *et al.* reported decreased MCP and CD59, and increased CFB and C3b in RPE cells treated with oxidised low-density lipoproteins [68,99]. Thurman *et al.* showed that combined treatment of stable RPE cell monolayers with hydrogen peroxide and complement-sufficient serum disrupts barrier function, as well as reducing surface expression of membrane-bound complement regulatory proteins. Addition of either component alone had no effect [100]. Furthermore, sublytic MAC activation on oxidatively stressed RPE cells induced polarised secretion of VEGF [100], the amount of which was later found to correlate with the degree of induced barrier disruption [101]. Numerous studies on mouse models of laser-induced CNV have also shown that VEGF expression is dependent on complement activation, particularly via the alternative pathway [21,22,59,102,103,104,105,106]. These findings implicate complement activation as an important upstream mediator of VEGF secretion in NV AMD.

Smoking is a well-known risk factor for AMD development and also associated with increased oxidative stress [2,107]. Wang *et al.* reported increased expression of C3a, C5, MAC and CFH in the RPE/choroid of mice with chronic cigarette exposure [108]. This was later to shown to be dependent on the alternative pathway of complement activation using a *CFB*−/− mouse model [109]. A recent study suggests smoking may activate complement via down-regulation of the antioxidant transcription factor Nrf2 [110]. Kunchithapautham *et al.* have additionally shown that mice exposed to cigarette smoke have increased retinal lipid deposition and that this is a complement-dependent process [111].

##### 3.3.2.2. Pro-Inflammatory Agents Produced in the Retina

Other groups have investigated the effects of pro-inflammatory agents that are generated at the retina. Zhou *et al.* found that photo-oxidised A2E, a bisretinoid lipofuscin pigment, can activate complement in human RPE cells *in vitro* [112] and that this is dependent on the alternative pathway of complement activation [113]. The authors further showed that pre-treatment of the RPE cells with vitamin E protected against photo-oxidation and complement activation [114]. Accumulation of photo-oxidised A2E in RPE cells has also been shown to decrease CFH expression [115]. Similarly, Ma *et al.* demonstrated that accumulation of intracellular A2E in cultured intraretinal microglia decreased CFH synthesis as well as increasing CFB production [116].

Chen *et al.* found that CFH synthesis by cultured RPE cells is also reduced by long-term treatment with oxidised photoreceptor outer segments [117]. More recently, Radu *et al.* showed that RPE cells with AMD-protective haplotypes, and not those with AMD-predisposing haplotypes, increased expression of CFH and other complement regulatory proteins when challenged with bisretinoid-containing *Abca4−/−* photoreceptor outer segments. This resulted in greater accumulation of C3/C3b and MAC on RPE cells [118].

Berchuck *et al.* showed that treating RPE cells with all-trans-retinal also results in decreased MCP and CD59 expression. Cell death was protected against by pre-treatment with the antioxidant resveratrol [119]. Hollyfield *et al.* immunised mice with carboxyethylpyrrole, an oxidation fragment of docosahexaenoic acid, which is an abundant fatty acid component of photoreceptor phospholipid membranes. These mice fixed C3 in Bruch’s membrane, accumulated sub-RPE drusen with age and developed GA-like lesions [120].

##### 3.3.2.3. Amyloid Beta

Amyloid beta (Abeta) is a major component of drusen and has been shown to co-localise with complement activation products within “amyloid vesicles” [121,122]. Wang *et al*. have found that Abeta binds CFI, inhibiting its ability to cleave and inactivate C3b [123]. The same group later showed that Abeta induced RPE cell production of monocyte chemoattractant protein-1, which can attract macrophages and microglia. It also increased TNFα and IL-1 beta production by macrophages and microglia, which up-regulated CFB production by RPE cells [124]. This is consistent with studies by Luo *et al.*, who reported that expression of complement components by RPE and microglial cells is influenced by pro-inflammatory cytokines [43], and that activated macrophages can induce CFB and C3 expression by RPE cells [125]. Furthermore, *CFH*−/− mice injected intraperitoneally with anti-Abeta antibody showed reduced retinal deposition of Abeta and activated C3 in a dose-dependent manner [126].

## 4. Systemic Complement Activation and AMD

Although the retina synthesises complement, the vast majority of circulating complement is produced by the liver. Complement deposited in the retina may therefore be of systemic origin. Several studies have investigated for an association between systemic complement dysregulation and AMD. The earliest was by Sivaprasad *et al.*, who found significantly raised plasma levels of C3a-desArg, the inactive form of the pro-inflammatory C3a anaphylatoxin, in AMD subjects compared with controls [23]. A later study also showed elevated plasma C3a-desArg levels in subjects with NV AMD, but not in those with the non-NV form [31]. In a more comprehensive analysis Scholl *et al.* found levels of all complement activation products to be significantly raised in AMD subjects, especially the activation split products Ba and C3d, which indicate chronic activation [24]. Similar findings were reported by both Reynolds *et al.* [26] and in a larger study by Hecker *et al.* [25]. Reynolds *et al.* found a significant association between the highest quartiles of Bb and C5a plasma levels and advanced AMD [26]. Hecker *et al.* showed that a one standard deviation change in CFD, CFB, Ba and C3d plasma levels was associated with an almost fivefold increased risk of AMD [25]. More recently, analysis of haemolytic complement assays showed significantly increased systemic activity of the alternative pathway, but not of the classical or lectin pathways in association with AMD [28]. Ristau *et al.* conducted the largest study to date (*n =* 2655) finding a significantly raised plasma C3d/C3 ratio in AMD subjects, implying chronic complement activation [30].

While collectively these studies implicate elevated systemic complement activity in AMD, several discrepancies exist. For instance, significantly reduced plasma levels of CFD were associated with AMD in a study by Silva *et al.* [29], whereas Stanton *et al.* found significantly raised CFD levels in AMD subjects [27]. Raised plasma levels of MAC in AMD subjects were reported by Scholl *et al.* [24], but not by Reynolds *et al.* [26] or Smailhodzic *et al.* [28]. Reynolds *et al.* suggested this may partly reflect the different proportions of AMD subtypes studied [26]. Indeed results from AMD subtype analyses, although inconsistent, suggest differential systemic complement activity. Scholl *et al.* showed significantly lower C3d levels in CNV subjects than in subjects with GA or early AMD [24]. In contrast to these findings Hecker *et al.* found a trend for greater increases in plasma levels of CFD, Ba and C3d in CNV subjects. A similar trend for greater increases in plasma CFD, CFB and Ba levels in GA subjects was observed, suggesting an association between AMD progression and systemic complement activation [25]. Reynolds *et al.* reported significantly higher plasma levels of Bb, C3a and C5a, and significantly lower plasma CFH levels in GA subjects specifically [26].

The effect of AMD-associated genetic variants on systemic complement activation is unclear. Hecker *et al.* found significantly raised plasma C3d levels in association with the single nucleotide polymorphism (SNP) rs2230199 in the *C3* gene, and lower C3d levels in association with the minor allele for SNP rs800292 in *CFH* [25]. Consistent with this, Ristau *et al.* showed associations between the same SNPS in *C3* and *CFH* with greater and lower C3d/C3 ratios respectively [30]. The risk genotype for SNP rs2230199 in *C3* has also been found to be associated with higher plasma levels of C5a [26], although this was not replicated by Hecker *et al.* [25]. The protective *CFB* genotype for SNP rs4151667 has been associated with a significantly lower plasma CFB level [28] and C3d/C3 ratio [30]. In addition, Reynolds *et al.* showed an inverse relationship between plasma CFH levels and AMD risk for subjects with the non-protective *CFB* genotype for this SNP [26].

Apart from these examples however, reported associations between genetic variants and systemic complement activation have not been reproduced. For example while Smailhodzic *et al.* found an association between systemic complement activity and the *CFH* Y402H polymorphism [28], this was not reproduced by six other studies [23,24,25,26,29,30]. Indeed a stronger correlation between systemic complement and AMD has been identified at the protein level rather than with genetic variation [24]. This may indicate that analysing by individual SNPs is too simplistic an approach and does not account for interactions between multiple genetic variants. Heurich *et al.* showed that combining risk variants of AMD-associated complement protein allotypes resulted in sixfold greater haemolytic activity compared with protective variants *in vitro* [77]. Analysis of haplotypes at *CFH* and *CFB-C2* loci has also yielded further genetic associations with systemic complement activation, further suggesting that polymorphisms may have greater functional consequences in combination than individually [24,25,30].

Alternatively, other factors may be more important in explaining systemic complement activation in AMD. Several groups found significantly increased systemic complement activation with advancing age [25,30,127], while others have shown gender-specific alterations in circulating complement levels [25,27,29]. High body mass index has been associated with increased complement activation [26,27], as well as a lower C3d/C3 ratio [30]. Ristau *et al.* also found a high C3d/C3 ratio was associated with smoking, whereas a low C3d/C3 ratio was associated with diabetes. Furthermore, the authors demonstrated in linear models that age, smoking status, gender, genetic polymorphisms and AMD status only explained <7% of the C3d/C3 ratio [30]. Gibson *et al.* similarly reported that only 8.8% of variation in plasma C1inh levels is attributable to age, gender, smoking, AMD status and *SERPING1* genotype [32]. The major factors underlying systemic complement activation in AMD therefore appear to be largely unknown.

## 5. Systemic *versus* Local Manipulation of Complement

Complement deposited in the retina could therefore be of either local or systemic origin, or both. The relative contribution from each source is yet to be determined. Insights into which is more important may, however, be indirectly inferred from studies of complement manipulation in AMD.

Delivery of complement-modulating compounds via either systemic or local routes has shown promising results in laser-induced CNV mouse models. Agents inhibiting C3a [102], C5a [102], C6 [21,104], CFB [22] and MAC formation [128,129] have been reported to inhibit CNV development, as can administration of the complement regulatory molecules CD59 [59,103,130] and CFH [57,105,131].

In studies by Bora *et al.* [59] and Liu *et al.* [104], complement-modulating compounds were administered both systemically and locally, allowing some comparison of the two modes of delivery. Bora *et al.* found that recombinant soluble mouse CD59a-IgG2a (rsCD59a-Fc) fusion protein inhibits development of CNV when injected either via intravitreal (50 µg) or intraperitoneal (100 µg) routes 24 hours before laser. The incidence of CNV in the group receiving intraperitoneal rsCD59a-Fc was 13% compared with 94% for PBS-injected controls. For mice receiving intravitreal rsCD59a-Fc the incidence of CNV was reduced to 30% compared with 93% in PBS-injected controls [59]. In the study by Liu *et al.*, mice either received eight daily intraperitoneal injections (50 µg) of anti-C6 antibody, with the last dose given immediately post-laser, or one subretinal dose (1.4 µg) immediately post-laser only. Compared with controls, CNV was inhibited by 77% and 73% at day three post-laser in mice receiving intraperitoneal and subretinal anti-C6 antibody injections respectively [104]. Although slightly greater inhibition of CNV development resulted from systemic rather than local administration in both studies, these differences were not reported as statistically significant. Furthermore, lower doses of these agents were required to achieve similar results when delivered locally.

Clinical trials of potential complement-modulating therapies for AMD have mostly been disappointing [132]. However, results from a phase II clinical trial last year showed reduced GA progression in patients receiving intravitreal injections of lampalizumab, an anti-CFD monoclonal antibody [133]. Earlier this year Roche initiated two phase III clinical trials, Chroma [134] and Spectri [135], investigating GA treatment with lampalizumab. No results are available as these studies are still enrolling patients. In contrast systemic administration of an anti-C5 monoclonal antibody, eculizumab, had no effect on GA progression, despite almost complete inhibition of systemic C5 activity. The authors suggested that systemic levels of eculizumab may not have been adequate to penetrate the RPE [136]. Consistent with this, the permeability of Bruch’s membrane to serum proteins has been shown to decrease with age [137] and a 50% decrease in choriocapillaris area has been observed underlying areas of GA [95]. It is conceivable that circulating complement-modulating therapies might reach retinal lesions more readily in NV AMD, where the blood-retinal barrier is breached. Indeed, Rohrer *et al.* demonstrated in a laser-induced mouse model that intravenously administered recombinant CFH (CR2-fH) reduced CNV size and localised to the RPE-choroid at sites of C3 deposition [57].

Eculizumab has also been investigated as a treatment for dense deposit disease (DDD) and C3 glomerulonephritis (C3GN). Similar to AMD, these renal diseases are associated with systemic complement dysregulation and glomerular C3 deposition [39]. Furthermore, patients with dense deposit disease often develop drusen [138,139]. Bomback *et al.* reported no change in drusen load or fundus autofluorescent pattern for two subjects with DDD/C3GN-related drusen, despite improvements in renal histology and laboratory parameters [140].

Another renal disease associated with complement-mediated glomerular damage is atypical haemolytic uraemic syndrome (aHUS). This too is associated with systemic complement activation and a number of genetic variants in complement-related genes, including *CFH*, *C3*, *CFI*, *CFB* and deletions in *CFHR1-3* [141,142]. The kidney produces similar complement components to the RPE-choroid [3]. While kidney transplant alone is often unsuccessful in aHUS associated with a *CFH* gene mutation, co-transplanting a liver without a pathogenic *CFH* variant can achieve favourable long-term outcomes [143]. Furthermore, aHUS has been reported to develop in a patient receiving a donor liver carrying a pathogenic *CFH* genetic variant [144]. It appears therefore that circulating rather than locally produced *CFH* determines aHUS risk. This contrasts with observations from liver transplantation in AMD however. Khandhadia *et al.* showed that while circulating CFH protein allotype is completely determined by donor liver *CFH* genotype, AMD risk in liver transplant patients is associated with recipient rather than donor *CFH* genotype [145]. These findings suggest that unlike aHUS, locally produced *CFH* plays a greater role than circulating *CFH* in AMD pathogenesis.

## 6. Conclusions

In summary, the retina expresses its own set of complement proteins, which may render it more susceptible to complement-mediated damage. Indeed complement dysregulation and altered complement production seem to occur locally with advancing age and in AMD. A growing body of evidence indicates that intraocular complement plays a central role in AMD pathogenesis, interacting with other known risk factors such as oxidative stress and pro-angiogenic growth factors. Furthermore, an association between systemic complement activation and AMD has also been firmly established. Retinal deposition of circulating complement components or defective complement regulatory proteins may contribute to disease.

Both locally and systemically produced complement could therefore play a role and compound one another in AMD. Determining the proportion of complement deposited in the retina that is locally or systemically produced might clarify which source is more important. This could potentially be directly investigated by analysing the relative proportions of complement protein allotypes in donor eyes from liver transplant patients with differing donor and recipient complement genotypes. Alternatively animal studies could determine whether tagged complement protein delivered systemically is deposited within the eye. Perhaps circulating complement may exert greater influence in NV AMD, where breakdown of the blood-retinal barrier affords greater access to the neuroretina. Despite these unanswered questions, current evidence from studies of local and systemic complement manipulation suggests intraocular delivery of novel complement-based therapies may prove more effective, particularly if the blood-retinal barriers are not compromised as is the case in early AMD and geographic atrophy.

## References

[1] Klein R., Peto T., Bird A., Vannewkirk M.R. (2004). The epidemiology of age-related macular degeneration. Am. J. Ophthalmol..

[2] Khandhadia S., Lotery A. (2010). Oxidation and age-related macular degeneration: Insights from molecular biology. Expert. Rev. Mol. Med..

[3] Anderson D.H., Radeke M.J., Gallo N.B., Chapin E.A., Johnson P.T., Curletti C.R., Hancox L.S., Hu J., Elbright J.N., Malek G. (2010). The pivotal role of the complement system in aging and age-related macular degeneration: Hypothesis re-visited. Prog. Retin. Eye Res..

[4] Hageman G.S., Luthert P.J., Victor Chong N.H., Johnson L.V., Anderson D.H., Mullins R.F. (2001). An integrated hypothesis that considers drusen as biomarkers of immune-mediated processes at the RPE-Bruch’s membrane interface in aging and age-related macular degeneration. Prog. Retin. Eye Res..

[5] Johnson L.V., Leitner W.P., Staples M.K., Anderson D.H. (2001). Complement activation and inflammatory processes in Drusen formation and age related macular degeneration. Exp. Eye Res..

[6] Mullins R.F., Aptsiauri N., Hageman G.S. (2001). Structure and composition of drusen associated with glomerulonephritis: Implications for the role of complement activation in drusen biogenesis. Eye (Lond).

[7] Crabb J.W., Miyagi M., Gu X., Shadrach K., West K.A., Sakaguchi H., Kamei M., Hasan A., Yan L., Rayborn M.E. (2002). Drusen proteome analysis: An approach to the etiology of age-related macular degeneration. Proc. Natl. Acad. Sci. USA.

[8] Mullins R.F., Russell S.R., Anderson D.H., Hageman G.S. (2000). Drusen associated with aging and age-related macular degeneration contain proteins common to extracellular deposits associated with atherosclerosis, elastosis, amyloidosis, and dense deposit disease. FASEB J..

[9] Hageman G.S., Anderson D.H., Johnson L.V., Hancox L.S., Taiber A.J., Hardisty L.I., Hageman J.L., Stockman H.A., Borchardt J.D., Gehrs K.M. (2005). A common haplotype in the complement regulatory gene factor H (HF1/CFH) predisposes individuals to age-related macular degeneration. Proc. Natl. Acad. Sci. USA.

[10] Edwards A.O., Ritter R., Abel K.J., Manning A., Panhuysen C., Farrer L.A. (2005). Complement factor H polymorphism and age-related macular degeneration. Science.

[11] Haines J.L., Hauser M.A., Schmidt S., Scott W.K., Olson L.M., Gallins P., Spencer K.L., Kwan S.Y., Noureddine M., Gilbert J.R. (2005). Complement factor H variant increases the risk of age-related macular degeneration. Science.

[12] Klein R.J., Zeiss C., Chew E.Y., Tsai J.Y., Sackler R.S., Haynes C., Henning A.K., SanGiovanni J.P., Mane S.M., Mayne S.T. (2005). Complement factor H polymorphism in age-related macular degeneration. Science.

[13] Gold B., Merriam J.E., Zernant J., Hancox L.S., Taiber A.J., Gehrs K., Cramer K., Neel J., Bergeron J., Barile G.R. (2006). Variation in factor B (BF) and complement component 2 (*C2*) genes is associated with age-related macular degeneration. Nat. Genet..

[14] Yates J.R., Sepp T., Matharu B.K., Khan J.C., Thurlby D.A., Shahid H., Clayton D.G., Hayward C., Morgan J., Wright A.F. (2007). Complement C3 variant and the risk of age-related macular degeneration. N. Engl. J. Med..

[15] Dinu V., Miller P.L., Zhao H. (2007). Evidence for association between multiple complement pathway genes and AMD. Genet. Epidemiol..

[16] Seddon J.M., Yu Y., Miller E.C., Reynolds R., Tan P.L., Gowrisankar S., Goldstein J.I., Triebwasser M., Anderson H.E., Zerbib J. (2013). Rare variants in CFI, C3 and C9 are associated with high risk of advanced age-related macular degeneration. Nat. Genet..

[17] Fagerness J.A., Maller J.B., Neale B.M., Reynolds R.C., Daly M.J., Seddon J.M. (2009). Variation near complement factor I is associated with risk of advanced AMD. Eur. J. Hum. Genet..

[18] Ennis S., Jomary C., Mullins R., Cree A., Chen X., Macleod A., Jones S., Collins A., Stone E., Lotery A. (2008). Association between the *SERPING1* gene and age-related macular degeneration: A two-stage case-control study. Lancet.

[19] Allikmets R., Dean M., Hageman G.S., Baird P.N., Klaver C.C., Bergen A.A., Weber B.H., International AMD Genetics Consortium (2009). The *SERPING1* gene and age-related macular degeneration. Lancet.

[20] Coffey P.J., Gias C., McDermott C.J., Lundh P., Pickering M.C., Sethi C., Bird A., Fitzke F.W., Maass A., Chen L.L. (2007). Complement factor H deficiency in aged mice causes retinal abnormalities and visual dysfunction. Proc. Natl. Acad. Sci. USA.

[21] Bora P.S., Sohn J.H., Cruz J.M., Jha P., Nishihori H., Wang Y., Kaliappan S., Kaplan H.J., Bora N.S. (2005). Role of complement and complement membrane attack complex in laser-induced choroidal neovascularization. J. Immunol..

[22] Bora N.S., Kaliappan S., Jha P., Xu Q., Sohn J.H., Dhaulakhandi D.B., Kaplan H.J., Bora P.S. (2006). Complement activation via alternative pathway is critical in the development of laser-induced choroidal neovascularization: Role of factor B and factor H. J. Immunol..

[23] Sivaprasad S., Adewoyin T., Bailey T.A., Dandekar S.S., Jenkins S., Webster A.R., Chong N.V. (2007). Estimation of systemic complement C3 activity in age-related macular degeneration. Arch. Ophthalmol..

[24] Scholl H.P., Charbel I.P., Walier M., Janzer S., Pollok-Kopp B., Borncke F., Fritsche L.G., Chong N.V., Fimmers R., Wienker T. (2008). Systemic complement activation in age-related macular degeneration. PLoS One.

[25] Hecker L.A., Edwards A.O., Ryu E., Tosakulwong N., Baratz K.H., Brown W.L., Charbel Issa P., Scholl H.P., Pollok-Kopp B., Schmid-Kubista K.E. (2010). Genetic control of the alternative pathway of complement in humans and age-related macular degeneration. Hum. Mol. Genet..

[26] Reynolds R., Hartnett M.E., Atkinson J.P., Giclas P.C., Rosner B., Seddon J.M. (2009). Plasma complement components and activation fragments: Associations with age-related macular degeneration genotypes and phenotypes. Invest Ophthalmol. Vis. Sci..

[27] Stanton C.M., Yates J.R., den Hollander A.I., Seddon J.M., Swaroop A., Stambolian D., Fauser S., Hoyng C., Yu Y., Atsuhiro K. (2011). Complement factor D in age-related macular degeneration. Invest Ophthalmol. Vis. Sci..

[28] Smailhodzic D., Klaver C.C., Klevering B.J., Boon C.J., Groenewoud J.M., Kirchhof B., Daha M.R., den Hollander A.I., Hoyng C.B. (2012). Risk alleles in *CFH* and *ARMS2* are independently associated with systemic complement activation in age-related macular degeneration. Ophthalmology.

[29] Silva A.S., Teixeira A.G., Bavia L., Lin F., Velletri R., Belfort R., Isaac L. (2012). Plasma levels of complement proteins from the alternative pathway in patients with age-related macular degeneration are independent of Complement Factor H Tyr(4)(0)(2)His polymorphism. Mol. Vis..

[30] Ristau T., Paun C., Ersoy L., Hahn M., Lechanteur Y., Hoyng C., de Jong E.K., Daha M.R., Kirchhof B., den Hollander A.I. (2014). Impact of the common genetic associations of age-related macular degeneration upon systemic complement component C3d levels. PLoS One.

[31] Machalinska A., Dziedziejko V., Mozolewska-Piotrowska K., Karczewicz D., Wiszniewska B., Machalinski B. (2009). Elevated plasma levels of C3a complement compound in the exudative form of age-related macular degeneration. Ophthalmic Res..

[32] Gibson J., Hakobyan S., Cree A.J., Collins A., Harris C.L., Ennis S., Morgan B.P., Lotery A.J. (2012). Variation in complement component C1 inhibitor in age-related macular degeneration. Immunobiology.

[33] Khandhadia S., Cipriani V., Yates J.R., Lotery A.J. (2012). Age-related macular degeneration and the complement system. Immunobiology.

[34] Dunkelberger J.R., Song W.C. (2010). Complement and its role in innate and adaptive immune responses. Cell Res..

[35] Walport M.J. (2001). Complement. First of two parts. N. Engl. J. Med..

[36] Walport M.J. (2001). Complement. Second of two parts. N. Engl. J. Med..

[37] Manderson A.P., Botto M., Walport M.J. (2004). The role of complement in the development of systemic lupus erythematosus. Annu. Rev. Immunol..

[38] Kavanagh D., Goodship T.H., Richards A. (2013). Atypical hemolytic uremic syndrome. Semin. Nephrol..

[39] Barbour T.D., Pickering M.C., Terence C.H. (2013). Dense deposit disease and C3 glomerulopathy. Semin. Nephrol..

[40] Gallenga C.E., Parmeggiani F., Costagliola C., Sebastiani A., Gallenga P.E. (2014). Inflammaging: Should this term be suitable for age related macular degeneration too?. Inflamm. Res..

[41] Xu H., Chen M., Forrester J.V. (2009). Para-inflammation in the aging retina. Prog. Retin. Eye Res..

[42] Morgan B.P., Gasque P. (1997). Extrahepatic complement biosynthesis: Where, when and why?. Clin. Exp. Immunol..

[43] Luo C., Chen M., Xu H. (2011). Complement gene expression and regulation in mouse retina and retinal pigment epithelium/choroid. Mol. Vis..

[44] Laufer J., Katz Y., Passwell J.H. (2001). Extrahepatic synthesis of complement proteins in inflammation. Mol. Immunol..

[45] Yanamadala V., Friedlander R.M. (2010). Complement in neuroprotection and neurodegeneration. Trends Mol. Med..

[46] Yu M., Zou W., Peachey N.S., McIntyre T.M., Liu J. (2012). A novel role of complement in retinal degeneration. Invest Ophthalmol. Vis. Sci..

[47] Hoh K.J., Lenassi E., Malik T.H., Pickering M.C., Jeffery G. (2013). Complement component C3 plays a critical role in protecting the aging retina in a murine model of age-related macular degeneration. Am. J. Pathol..

[48] Seth A., Cui J., To E., Kwee M., Matsubara J. (2008). Complement-associated deposits in the human retina. Invest Ophthalmol. Vis. Sci..

[49] Mandal M.N., Ayyagari R. (2006). Complement factor H: Spatial and temporal expression and localization in the eye. Invest Ophthalmol. Vis. Sci..

[50] Chen H., Liu B., Lukas T.J., Neufeld A.H. (2008). The aged retinal pigment epithelium/choroid: A potential substratum for the pathogenesis of age-related macular degeneration. PLoS One.

[51] Chen M., Muckersie E., Forrester J.V., Xu H. (2010). Immune activation in retinal aging: A gene expression study. Invest Ophthalmol. Vis. Sci..

[52] Faber C., Williams J., Juel H.B., Greenwood J., Nissen M.H., Moss S.E. (2012). Complement factor H deficiency results in decreased neuroretinal expression of Cd59a in aged mice. Invest Ophthalmol. Vis. Sci..

[53] Ma W., Cojocaru R., Gotoh N., Gieser L., Villasmil R., Cogliati T., Swaroop A., Wong W.T. (2013). Gene expression changes in aging retinal microglia: Relationship to microglial support functions and regulation of activation. Neurobiol. Aging.

[54] Rutar M., Valter K., Natoli R., Provis J.M. (2014). Synthesis and propagation of complement C3 by microglia/monocytes in the aging retina. PLoS One.

[55] Lohr H.R., Kuntchithapautham K., Sharma A.K., Rohrer B. (2006). Multiple, parallel cellular suicide mechanisms participate in photoreceptor cell death. Exp. Eye Res..

[56] Rohrer B., Guo Y., Kunchithapautham K., Gilkeson G.S. (2007). Eliminating complement factor D reduces photoreceptor susceptibility to light-induced damage. Invest Ophthalmol. Vis. Sci..

[57] Rohrer B., Long Q., Coughlin B., Wilson R.B., Huang Y., Qiao F., Tang P.H., Kunchithapautham K., Gilkeson G.S., Tomlinson S. (2009). A targeted inhibitor of the alternative complement pathway reduces angiogenesis in a mouse model of age-related macular degeneration. Invest Ophthalmol. Vis. Sci..

[58] Lyzogubov V.V., Tytarenko R.G., Jha P., Liu J., Bora N.S., Bora P.S. (2010). Role of ocular complement factor H in a murine model of choroidal neovascularization. Am. J. Pathol..

[59] Bora N.S., Kaliappan S., Jha P., Xu Q., Sivasankar B., Harris C.L., Morgan B.P., Bora P.S. (2007). CD59, a complement regulatory protein, controls choroidal neovascularization in a mouse model of wet-type age-related macular degeneration. J. Immunol..

[60] Baudouin C., Peyman G.A., Fredj-Reygrobellet D., Gordon W.C., Lapalus P., Gastaud P., Bazan N.G. (1992). Immunohistological study of subretinal membranes in age-related macular degeneration. Jpn. J. Ophthalmol..

[61] Lommatzsch A., Hermans P., Weber B., Pauleikhoff D. (2007). Complement factor H variant Y402H and basal laminar deposits in exudative age-related macular degeneration. Graefes Arch. Clin. Exp. Ophthalmol..

[62] Lommatzsch A., Hermans P., Muller K.D., Bornfeld N., Bird A.C., Pauleikhoff D. (2008). Are low inflammatory reactions involved in exudative age-related macular degeneration? Morphological and immunhistochemical analysis of AMD associated with basal deposits. Graefes Arch. Clin. Exp. Ophthalmol..

[63] Yuan X., Gu X., Crabb J.S., Yue X., Shadrach K., Hollyfield J.G., Crabb J.W. (2010). Quantitative proteomics: Comparison of the macular Bruch membrane/choroid complex from age-related macular degeneration and normal eyes. Mol. Cell Proteomics.

[64] Loyet K.M., Deforge L.E., Katschke K.J., Diehl L., Graham R.R., Pao L., Sturgeon L., Lewin-Koh S.C., Hollyfield J.G., van Lookeren Campagne M. (2012). Activation of the alternative complement pathway in vitreous is controlled by genetics in age-related macular degeneration. Invest Ophthalmol. Vis. Sci..

[65] Mullins R.F., Dewald A.D., Streb L.M., Wang K., Kuehn M.H., Stone E.M. (2011). Elevated membrane attack complex in human choroid with high risk complement factor H genotypes. Exp. Eye Res..

[66] Mullins R.F., Faidley E.A., Daggett H.T., Jomary C., Lotery A.J., Stone E.M. (2009). Localization of complement 1 inhibitor (C1INH/SERPING1) in human eyes with age-related macular degeneration. Exp. Eye Res..

[67] Vogt S.D., Curcio C.A., Wang L., Li C.M., McGwin G., Medeiros N.E., Philp N.J., Kimble J.A., Read R.W. (2011). Retinal pigment epithelial expression of complement regulator CD46 is altered early in the course of geographic atrophy. Exp. Eye Res..

[68] Ebrahimi K.B., Fijalkowski N., Cano M., Handa J.T. (2013). Decreased membrane complement regulators in the retinal pigmented epithelium contributes to age-related macular degeneration. J. Pathol..

[69] Weismann D., Hartvigsen K., Lauer N., Bennett K.L., Scholl H.P., Charbel I.P., Cano M., Brandstätter H., Tsimikas S., Skerka C. (2011). Complement factor H binds malondialdehyde epitopes and protects from oxidative stress. Nature.

[70] Johnson P.T., Betts K.E., Radeke M.J., Hageman G.S., Anderson D.H., Johnson L.V. (2006). Individuals homozygous for the age-related macular degeneration risk-conferring variant of complement factor H have elevated levels of CRP in the choroid. Proc. Natl. Acad. Sci. USA.

[71] Shaw P.X., Zhang L., Zhang M., Du H., Zhao L., Lee C., Grob S., Lim S.L., Hughes G., Lee J. (2012). Complement factor H genotypes impact risk of age-related macular degeneration by interaction with oxidized phospholipids. Proc. Natl. Acad. Sci. USA.

[72] Perkins S.J., Nan R., Li K., Khan S., Miller A. (2012). Complement factor H-ligand interactions: Self-association, multivalency and dissociation constants. Immunobiology.

[73] Pangburn M.K. (2000). Host recognition and target differentiation by factor H, a regulator of the alternative pathway of complement. Immunopharmacology.

[74] Zipfel P.F., Skerka C., Hellwage J., Jokiranta S.T., Meri S., Brade V., Kraiczy P., Noris M., Remuzzi G. (2002). Factor H family proteins: On complement, microbes and human diseases. Biochem. Soc. Trans..

[75] Montes T., Tortajada A., Morgan B.P., Rodriguez de C.S., Harris C.L. (2009). Functional basis of protection against age-related macular degeneration conferred by a common polymorphism in complement factor B. Proc. Natl. Acad. Sci. USA.

[76] Tortajada A., Montes T., Martinez-Barricarte R., Morgan B.P., Harris C.L., de Cordoba S.R. (2009). The disease-protective complement factor H allotypic variant Ile62 shows increased binding affinity for C3b and enhanced cofactor activity. Hum. Mol. Genet..

[77] Heurich M., Martinez-Barricarte R., Francis N.J., Roberts D.L., Rodriguez de C.S., Morgan B.P., Harris C.L. (2011). Common polymorphisms in C3, factor B, and factor H collaborate to determine systemic complement activity and disease risk. Proc. Natl. Acad. Sci. USA.

[78] Giannakis E., Jokiranta T.S., Male D.A., Ranganathan S., Ormsby R.J., Fischetti V.A., Mold C., Gordon D.L. (2003). A common site within factor H SCR 7 responsible for binding heparin, C-reactive protein and streptococcal M protein. Eur. J. Immunol..

[79] Clark S.J., Perveen R., Hakobyan S., Morgan B.P., Sim R.B., Bishop P.N., Day A.J. (2010). Impaired binding of the age-related macular degeneration-associated complement factor H 402H allotype to Bruch’s membrane in human retina. J. Biol. Chem..

[80] Kelly U., Yu L., Kumar P., Ding J.D., Jiang H., Hageman G.S., Arshavsky V.Y., Frank M.M., Hauser M.A., Rickman C.B. (2010). Heparan sulfate, including that in Bruch’s membrane, inhibits the complement alternative pathway: Implications for age-related macular degeneration. J. Immunol..

[81] Skerka C., Lauer N., Weinberger A.A., Keilhauer C.N., Suhnel J., Smith R., Schlotzer-Schrehardt U., Fritsche L., Heinen S., Hartmann A. (2007). Defective complement control of factor H (Y402H) and FHL-1 in age-related macular degeneration. Mol. Immunol..

[82] Lauer N., Mihlan M., Hartmann A., Schlotzer-Schrehardt U., Keilhauer C., Scholl H.P., Charbel Issa P., Holz F., Weber B.H., Skerka C. (2011). Complement regulation at necrotic cell lesions is impaired by the age-related macular degeneration-associated factor-H His402 risk variant. J. Immunol..

[83] Ormsby R.J., Ranganathan S., Tong J.C., Griggs K.M., Dimasi D.P., Hewitt A.W., Burdon K.P., Craig J.E., Hoh J., Gordon D.L. (2008). Functional and structural implications of the complement factor H Y402H polymorphism associated with age-related macular degeneration. Invest Ophthalmol. Vis. Sci..

[84] Laine M., Jarva H., Seitsonen S., Haapasalo K., Lehtinen M.J., Lindeman N., Anderson D.H., Johnson P.T., Jarvela I., Jokiranta T.S. (2007). Y402H polymorphism of complement factor H affects binding affinity to C-reactive protein. J. Immunol..

[85] Herbert A.P., Deakin J.A., Schmidt C.Q., Blaum B.S., Egan C., Ferreira V.P., Pangburn M.K., Lyon M., Uhrin D., Barlow P.N. (2007). Structure shows that a glycosaminoglycan and protein recognition site in factor H is perturbed by age-related macular degeneration-linked single nucleotide polymorphism. J. Biol. Chem..

[86] Sjoberg A.P., Trouw L.A., Clark S.J., Sjolander J., Heinegard D., Sim R.B., Day A.J., Blom A.M. (2007). The factor H variant associated with age-related macular degeneration (His-384) and the non-disease-associated form bind differentially to C-reactive protein, fibromodulin, DNA, and necrotic cells. J. Biol. Chem..

[87] Yu J., Wiita P., Kawaguchi R., Honda J., Jorgensen A., Zhang K., Fischetti V.A., Sun H. (2007). Biochemical analysis of a common human polymorphism associated with age-related macular degeneration. Biochemistry.

[88] Okemefuna A.I., Nan R., Miller A., Gor J., Perkins S.J. (2010). Complement factor H binds at two independent sites to C-reactive protein in acute phase concentrations. J. Biol. Chem..

[89] Black S., Kushner I., Samols D. (2004). C-reactive Protein. J. Biol. Chem..

[90] Yeh E.T. (2004). CRP as a mediator of disease. Circulation.

[91] Hong T., Tan A.G., Mitchell P., Wang J.J. (2011). A review and meta-analysis of the association between C-reactive protein and age-related macular degeneration. Surv. Ophthalmol..

[92] Seddon J.M., George S., Rosner B., Rifai N. (2005). Progression of age-related macular degeneration: Prospective assessment of C-reactive protein, interleukin 6, and other cardiovascular biomarkers. Arch. Ophthalmol..

[93] Robman L., Baird P.N., Dimitrov P.N., Richardson A.J., Guymer R.H. (2010). C-reactive protein levels and complement factor H polymorphism interaction in age-related macular degeneration and its progression. Ophthalmology.

[94] Bhutto I.A., Baba T., Merges C., Juriasinghani V., McLeod D.S., Lutty G.A. (2011). C-reactive protein and complement factor H in aged human eyes and eyes with age-related macular degeneration. Br. J. Ophthalmol..

[95] McLeod D.S., Grebe R., Bhutto I., Merges C., Baba T., Lutty G.A. (2009). Relationship between RPE and choriocapillaris in age-related macular degeneration. Invest Ophthalmol. Vis. Sci..

[96] Suzuki M., Kamei M., Itabe H., Yoneda K., Bando H., Kume N., Tano Y. (2007). Oxidized phospholipids in the macula increase with age and in eyes with age-related macular degeneration. Mol. Vis..

[97] Wu Z., Lauer T.W., Sick A., Hackett S.F., Campochiaro P.A. (2007). Oxidative stress modulates complement factor H expression in retinal pigmented epithelial cells by acetylation of FOXO3. J. Biol. Chem..

[98] Lau L.I., Chiou S.H., Liu C.J., Yen M.Y., Wei Y.H. (2011). The effect of photo-oxidative stress and inflammatory cytokine on complement factor H expression in retinal pigment epithelial cells. Invest Ophthalmol. Vis. Sci..

[99] Ebrahimi K.B., Fijalkowski N., Cano M., Handa J.T. (2014). Oxidized Low-Density-Lipoprotein-Induced Injury in Retinal Pigment Epithelium Alters Expression of the Membrane Complement Regulatory Factors CD46 and CD59 through Exosomal and Apoptotic Bleb Release. Adv. Exp. Med. Biol..

[100] Thurman J.M., Renner B., Kunchithapautham K., Ferreira V.P., Pangburn M.K., Ablonczy Z., Tomlinson S., Holers V.M., Rohrer B. (2009). Oxidative stress renders retinal pigment epithelial cells susceptible to complement-mediated injury. J. Biol. Chem..

[101] Bandyopadhyay M., Rohrer B. (2012). Matrix metalloproteinase activity creates pro-angiogenic environment in primary human retinal pigment epithelial cells exposed to complement. Invest Ophthalmol. Vis. Sci..

[102] Nozaki M., Raisler B.J., Sakurai E., Sarma J.V., Barnum S.R., Lambris J.D., Chen Y., Zhang K., Ambati B.K., Baffi J.Z. (2006). Drusen complement components C3a and C5a promote choroidal neovascularization. Proc. Natl. Acad. Sci. USA.

[103] Bora N.S., Jha P., Lyzogubov V.V., Kaliappan S., Liu J., Tytarenko R.G., Fraser D.A., Morgan B.P., Bora P.S. (2010). Recombinant membrane-targeted form of CD59 inhibits the growth of choroidal neovascular complex in mice. J. Biol. Chem..

[104] Liu J., Jha P., Lyzogubov V.V., Tytarenko R.G., Bora N.S., Bora P.S. (2011). Relationship between complement membrane attack complex, chemokine (C-C motif) ligand 2 (CCL2) and vascular endothelial growth factor in mouse model of laser-induced choroidal neovascularization. J. Biol. Chem..

[105] Rohrer B., Coughlin B., Bandyopadhyay M., Holers V.M. (2012). Systemic human CR2-targeted complement alternative pathway inhibitor ameliorates mouse laser-induced choroidal neovascularization. J. Ocul. Pharmacol. Ther..

[106] Rohrer B., Coughlin B., Kunchithapautham K., Long Q., Tomlinson S., Takahashi K., Holers V.M. (2011). The alternative pathway is required, but not alone sufficient, for retinal pathology in mouse laser-induced choroidal neovascularization. Mol. Immunol..

[107] Smith W., Assink J., Klein R., Mitchell P., Klaver C.C., Klein B.E., Hofman A., Jensen S., Wang J.J., de Jong P.T. (2001). Risk factors for age-related macular degeneration: Pooled findings from three continents. Ophthalmology.

[108] Wang A.L., Lukas T.J., Yuan M., Du N., Handa J.T., Neufeld A.H. (2009). Changes in retinal pigment epithelium related to cigarette smoke: Possible relevance to smoking as a risk factor for age-related macular degeneration. PLoS One.

[109] Woodell A., Coughlin B., Kunchithapautham K., Casey S., Williamson T., Ferrell W.D., Atkinson C., Jones B.W., Rohrer B. (2013). Alternative complement pathway deficiency ameliorates chronic smoke-induced functional and morphological ocular injury. PLoS One.

[110] Wang L., Kondo N., Cano M., Ebrahimi K., Yoshida T., Barnett B.P., Biswal S., Handa J.T. (2014). Nrf2 signaling modulates cigarette smoke-induced complement activation in retinal pigmented epithelial cells. Free Radic. Biol. Med..

[111] Kunchithapautham K., Atkinson C., Rohrer B. (2014). Smoke-exposure causes endoplasmic reticulum stress and lipid accumulation in retinal pigment epithelium through oxidative stress and complement activation. J. Biol. Chem..

[112] Zhou J., Jang Y.P., Kim S.R., Sparrow J.R. (2006). Complement activation by photooxidation products of A2E, a lipofuscin constituent of the retinal pigment epithelium. Proc. Natl. Acad. Sci. USA.

[113] Zhou J., Kim S.R., Westlund B.S., Sparrow J.R. (2009). Complement activation by bisretinoid constituents of RPE lipofuscin. Invest Ophthalmol. Vis. Sci..

[114] Sparrow J.R., Ueda K., Zhou J. (2012). Complement dysregulation in AMD: RPE-Bruch’s membrane-choroid. Mol. Aspects Med..

[115] Bian Q., Gao S., Zhou J., Qin J., Taylor A., Johnson E.J., Tang G., Sparrow J.R., Gierhart D., Shang F. (2012). Lutein and zeaxanthin supplementation reduces photooxidative damage and modulates the expression of inflammation-related genes in retinal pigment epithelial cells. Free Radic. Biol. Med..

[116] Ma W., Coon S., Zhao L., Fariss R.N., Wong W.T. (2013). A2E accumulation influences retinal microglial activation and complement regulation. Neurobiol. Aging.

[117] Chen M., Forrester J.V., Xu H. (2007). Synthesis of complement factor H by retinal pigment epithelial cells is down-regulated by oxidized photoreceptor outer segments. Exp. Eye Res..

[118] Radu R.A., Hu J., Jiang Z., Bok D. (2014). Bisretinoid-mediated complement activation on retinal pigment epithelial cells is dependent on complement factor H haplotype. J. Biol. Chem..

[119] Berchuck J.E., Yang P., Toimil B.A., Ma Z., Baciu P., Jaffe G.J. (2013). All-trans-retinal sensitizes human RPE cells to alternative complement pathway-induced cell death. Invest Ophthalmol. Vis. Sci..

[120] Hollyfield J.G., Bonilha V.L., Rayborn M.E., Yang X., Shadrach K.G., Lu L., Ufret R.L., Salomon R.G., Perez V.L. (2008). Oxidative damage-induced inflammation initiates age-related macular degeneration. Nat. Med..

[121] Isas J.M., Luibl V., Johnson L.V., Kayed R., Wetzel R., Glabe C.G., Langen R., Chen J. (2010). Soluble and mature amyloid fibrils in drusen deposits. Invest Ophthalmol. Vis. Sci..

[122] Johnson L.V., Leitner W.P., Rivest A.J., Staples M.K., Radeke M.J., Anderson D.H. (2002). The Alzheimer’s A beta -peptide is deposited at sites of complement activation in pathologic deposits associated with aging and age-related macular degeneration. Proc. Natl. Acad. Sci. USA.

[123] Wang J., Ohno-Matsui K., Yoshida T., Kojima A., Shimada N., Nakahama K., Safranova O., Iwata N., Saido T.C., Mochizuki M. (2008). Altered function of factor I caused by amyloid beta: Implication for pathogenesis of age-related macular degeneration from Drusen. J. Immunol..

[124] Wang J., Ohno-Matsui K., Yoshida T., Shimada N., Ichinose S., Sato T., Mochizuki M., Morita I. (2009). Amyloid-beta up-regulates complement factor B in retinal pigment epithelial cells through cytokines released from recruited macrophages/microglia: Another mechanism of complement activation in age-related macular degeneration. J. Cell Physiol..

[125] Luo C., Zhao J., Madden A., Chen M., Xu H. (2013). Complement expression in retinal pigment epithelial cells is modulated by activated macrophages. Exp. Eye Res..

[126] Catchpole I., Germaschewski V., Hoh K.J., Lundh von L.P., Ford S., Gough G., Adamson P., Overend P., Hilpert J., Lopez F.J. (2013). Systemic administration of Abeta mAb reduces retinal deposition of Abeta and activated complement C3 in age-related macular degeneration mouse model. PLoS One.

[127] Hakobyan S., Harris C.L., Tortajada A., Goicochea de J.E., Garcia-Layana A., Fernandez-Robredo P., Rodriguez de Cordoba S., Morgan B.P. (2008). Measurement of factor H variants in plasma using variant-specific monoclonal antibodies: Application to assessing risk of age-related macular degeneration. Invest Ophthalmol. Vis. Sci..

[128] Lipo E., Cashman S.M., Kumar-Singh R. (2013). Aurintricarboxylic acid inhibits complement activation, membrane attack complex, and choroidal neovascularization in a model of macular degeneration. Invest Ophthalmol. Vis. Sci..

[129] Birke M.T., Lipo E., Adhi M., Birke K., Kumar-Singh R. (2014). AAV-mediated expression of human PRELP inhibits complement activation, choroidal neovascularization and deposition of membrane attack complex in mice. Gene Ther..

[130] Cashman S.M., Ramo K., Kumar-Singh R. (2011). A non membrane-targeted human soluble CD59 attenuates choroidal neovascularization in a model of age related macular degeneration. PLoS One.

[131] Kim S.J., Kim J., Lee J., Cho S.Y., Kang H.J., Kim K.Y., Jin D.K. (2013). Intravitreal human complement factor H in a rat model of laser-induced choroidal neovascularisation. Br. J. Ophthalmol..

[132] Weber B.H., Charbel I.P., Pauly D., Herrmann P., Grassmann F., Holz F.G. (2014). The role of the complement system in age-related macular degeneration. Dtsch. Arztebl. Int..

[133] Hetwick C.C. MAHALO Finds New Biomarker for Dry Macular Degeneration, Medscape Medical News 21 November 2013. http://www.medscape.com/viewarticle/814822.

[134] Hoffmann-La Roche A Study Investigating the Safety and Efficacy of Lampalizumab Intravitreal Injections in Patients with Geographic Atrophy Secondary to Age-Related Macular Degeneration (CHROMA). https://clinicaltrials.gov/ct2/show/NCT02247479.

[135] Hoffmann-La Roche A Study Investigating the Safety and Efficacy of Lampalizumab Intravitreal Injections in Patients with Geographic Atrophy Secondary to Age-Related Macular Degeneration (SPECTRI). https://clinicaltrials.gov/ct2/show/NCT02247531.

[136] Yehoshua Z., de Amorim Garcia Filho C.A., Nunes R.P., Gregori G., Penha F.M., Moshfeghi A.A., Zhang K., Sadda S., Feuer W., Rosenfeld P.J. (2014). Systemic complement inhibition with eculizumab for geographic atrophy in age-related macular degeneration: The COMPLETE study. Ophthalmology.

[137] Moore D.J., Clover G.M. (2001). The effect of age on the macromolecular permeability of human Bruch’s membrane. Invest. Ophthalmol. Vis. Sci..

[138] D’souza Y.B., Jones C.J., Short C.D., Roberts I.S., Bonshek R.E. (2009). Oligosaccharide composition is similar in drusen and dense deposits in membranoproliferative glomerulonephritis type II. Kidney Int..

[139] McAvoy C.E., Silvestri G. (2005). Retinal changes associated with type 2 glomerulonephritis. Eye (Lond).

[140] Bomback A.S., Smith R.J., Barile G.R., Zhang Y., Heher E.C., Herlitz L., Stokes M.B., Markowitz G.S., D’Agati V.D., Canetta P.A. (2012). Eculizumab for dense deposit disease and C3 glomerulonephritis. Clin. J. Am. Soc. Nephrol..

[141] Noris M., Caprioli J., Bresin E., Mossali C., Pianetti G., Gamba S., Daina E., Fenili C., Castelletti F., Sorosina A. (2010). Relative role of genetic complement abnormalities in sporadic and familial aHUS and their impact on clinical phenotype. Clin. J. Am. Soc. Nephrol..

[142] Geerdink L.M., Westra D., van Wijk J.A., Dorresteijn E.M., Lilien M.R., Davin J.C., Komhoff M., Van Hoeck K., van der Vlugt A., van den Heuvel L.P. (2012). Atypical hemolytic uremic syndrome in children: Complement mutations and clinical characteristics. Pediatr. Nephrol..

[143] Saland J.M., Ruggenenti P., Remuzzi G. (2009). Liver-kidney transplantation to cure atypical hemolytic uremic syndrome. J. Am. Soc. Nephrol..

[144] Brown J.H., Tellez J., Wilson V., Mackie I.J., Scully M., Tredger M.M., Moore I., McDougall N.I., Strain L., Marchbank K.J. (2012). Postpartum aHUS secondary to a genetic abnormality in factor H acquired through liver transplantation. Am. J. Transplant..

[145] Khandhadia S., Hakobyan S., Heng L.Z., Gibson J., Adams D.H., Alexander G.J., Gibson J.M., Martin K.R., Menon G., Nask K. (2013). Age-related macular degeneration and modification of systemic complement factor H production through liver transplantation. Ophthalmology.

